# Vitronectin Levels in the Plasma of Neuroblastoma Patients and Culture Media of 3D Models: A Prognostic Circulating Biomarker?

**DOI:** 10.3390/ijms25168733

**Published:** 2024-08-10

**Authors:** Amparo López-Carrasco, Isaac Vieco-Martí, Sofía Granados-Aparici, Delia Acevedo-León, Nuria Estañ-Capell, Raquel Portugal, Jorge Huerta-Aragonés, Adela Cañete, Samuel Navarro, Rosa Noguera

**Affiliations:** 1 Incliva Biomedical Health Research Institute, 46010 Valencia, Spain; amparolopezcarrasco@gmail.com (A.L.-C.); sograap@gmail.com (S.G.-A.); samuel.navarro@uv.es (S.N.); 2 CIBER of Cancer (CIBERONC), 28029 Madrid, Spain; 3 University Hospital Doctor Peset, 46017 Valencia, Spain; 4 General University Hospital of Burgos, 09006 Burgos, Spain; rportugal@saludcastillayleon.es; 5 General University Hospital Gregorio Marañón, 28007 Madrid, Spain; 6 Politechnic and University Hospital La Fe, 46026 Valencia, Spain; 7 Pathology Department, Medical School, University of Valencia, 46010 Valencia, Spain

**Keywords:** pediatric cancer, liquid biopsy, precision medicine, hydrogels, extracellular matrix

## Abstract

Vitronectin is a glycoprotein present in plasma and the extracellular matrix that is implicated in cell migration. The high amount of vitronectin found in neuroblastoma biopsies has been associated with poor prognosis. Moreover, increased vitronectin levels have been described in the plasma of patients with different cancers. Our aim was to assess vitronectin as a potential circulating biomarker of neuroblastoma prognosis. Vitronectin concentration was quantified using ELISA in culture media of four neuroblastoma cell lines grown in a monolayer and in 3D models, and in the plasma of 114 neuroblastoma patients. Three of the neuroblastoma cell lines secreted vitronectin to culture media when cultured in a monolayer and 3D models. Vitronectin release was higher by neuroblastoma cells cultured in 3D models than in the monolayer and was still elevated when cells were grown in 3D scaffolds with cross-linked vitronectin. Vitronectin secretion occurred independently of cell numbers in cultures. Its concentration in the plasma of neuroblastoma patients ranged between 52.4 and 870 µg/mL (median, 218 µg/mL). A ROC curve was used to establish a cutoff of 361 µg/mL, above which patients over 18 months old had worse prognosis (*p* = 0.0018). Vitronectin could be considered a new plasma prognostic biomarker in neuroblastoma and warrants confirmation in collaborative studies. Drugs inhibiting vitronectin interactions with cells and/or the extracellular matrix could represent a significant improvement in survival for neuroblastoma patients.

## 1. Introduction

Vitronectin (VN), also known as serum spreading factor, is a multifunctional glycoprotein present in plasma and the extracellular matrix (ECM) [1]. It contains multiple cell receptor binding sites including integrins, urokinase-type plasminogen activator receptor (uPAR), and plasminogen activator inhibitor-1 (PAI-1) [2]. VN seems to anchor to ECM fibers and proteoglycans, leading to transitory ECM element–cell junctions, cell adhesion, and migration [3,4,5]. Indeed, coating culture surfaces with VN have been used for many years to promote the adhesion and growth of stem cells in their undifferentiated state and to direct differentiation [6,7]. Besides its role in spreading and metastasis, VN is involved in other key steps of cancer such as apoptosis, inflammation, vascular permeability, and vascular endothelial growth factor-induced angiogenesis [8,9,10]. Its association with tumor aggressiveness has been described in several cancers [8,11,12,13,14,15,16,17], including neuroblastoma (NB) [5].

NB originates from the neural crest in the sympathetic nervous system and is one of the most common pediatric solid tumors [18]. The risk of progression in NB patients is defined by several clinical, biological and genetic features [19], and the current survival rate of high-risk (HR) NB is under 50% [20]. Anchoring molecules, such as VN, could become primary targets for pharmacological strategies to increase this survival rate, as has occurred in other cancers [21]. In previous studies, we detected an elevated amount of VN in the cytoplasm of malignant neuroblasts and, adjacent to them, applied immunohistochemistry (IHC) on NB biopsies, which was related to poor outcome of HR-NB [5]. Moreover, we observed that this VN secreted to ECM form tracks, postulating that VN could participate in tumor cell migration [22]. In subsequent studies, our experimental models (orthotopic xenograft VN knock-out mice, and two 3D models: hydrogels (HGs) of methacrylated gelatin plus increasing concentrations of methacrylated alginate, and HGs of polyethylene glycol with and without cross-linked VN) supported the role of this glycoprotein in NB cell dynamics, aggressiveness, and in the clonal selection of segmental chromosomal aberrations (SCAs) [23,24,25,26].

Besides the role of VN in tumor ECM, its presence in blood plasma and in other types of liquid biopsies (ascites and cerebrospinal fluid) has also been studied in several tumor types. Specifically, elevated concentrations of circulating VN have been observed in adult patients with glioma, melanoma, breast, ovarian, endometrium cancer, and in pediatric patients with Hodgkin lymphoma and acute lymphoblastic leukemia [12,14,15,16,27,28,29,30,31,32,33]. VN in plasma is particularly understudied in pediatric solid tumors, including NB [34]. These tumors represent a great clinical challenge due to their aggressiveness and high mortality, and, in many cases, to the difficulty of obtaining appropriate biopsies, given the young age of some patients and the high frequency of intratumor heterogeneity [35].

The aim of the present study was to explore the value of VN detection in liquid biopsy as a novel diagnostic, prognostic, and therapeutic biomarker. We evaluated VN secretion to culture media by different NB cell lines grown in 2D and 3D HGs, and determined whether VN secretion was reflected in the plasma of HR-NB patients.

## 2. Results

### 2.1. Higher VN Release in 3D Models

**Figure 1:**
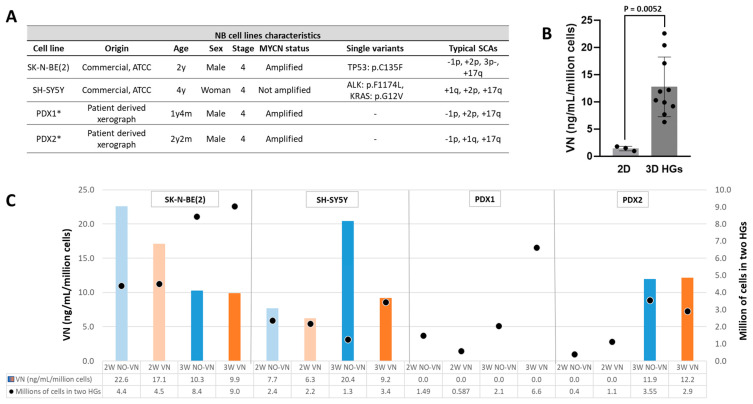
VN secretion by NB cells to culture media. (**A**) Origin and main clinical-molecular characteristics of the four NB cell lines. All presented aggressive features as derived from stage 4 patients, being *MYCN*-amplified or *ALK*-mutated and having SCAs. * For more details of PDX origin, see ref. [36]. (**B**) Comparison of VN levels secreted to culture media of 2D (monolayer) and 3D HG cultures by NB cell lines in which VN detection was positive by ELISA (*p*-value = 0.0052). (**C**) Concentration of VN secreted by the four NB cell lines to culture media of the 3D HGs in ng/mL per million cells (left Y-axis scale); blue and orange bars represent HGs without (NO-VN) and with cross-linked VN (VN), respectively; light colors refer to 2 weeks (2W) of culture and dark colors to 3W; number of cells calculated with digital analysis in two HGs from which we collected the culture media, measured in millions of cells, as shown on the right Y-axis scale (dots inside the bars).

We studied the VN levels in culture media of four NB cell lines (Figure 1A) grown in 2D until reaching confluence and in HGs for two and three weeks. VN release in control HGs (no cells) with cross-linked VN was null at both culture times. However, all but one cell line secreted VN to culture media when grown in 2D and in 3D models. SK-N-BE(2) and SH-SY5Y cell lines secreted VN in all growth conditions. PDX2 showed VN secretion when cultured in 2D and in HGs after three weeks of culture. We were unable to detect VN in culture media of PDX1 in any of the culture conditions. Aside from the abovementioned negative cases, VN concentration was significantly higher in media of the 3D models (median = 11.1 ng/mL/million cells) than in monolayer (median = 1.5 ng/mL/million cells) cultures (*p* = 0.0052, Figure 1B).

### 2.2. VN Secretion Independent of Cell Numbers

Pearson’s r correlation showed that the VN concentration secretion to culture media per million of cells and the estimated number of cells in HGs by digital analysis were independent variants. SK-N-BE(2) presented a marked reduction in VN secretion to the culture media after three weeks of culture (mean = 10.1 ng/mL/million cells) compared to two weeks (mean = 19.9 ng/mL/million cells), despite the greater number of cells estimated in the HGs with longer time. The opposite was observed for the SH-SY5Y (mean = 14.2 ng/mL at 3 weeks vs. 7 ng/mL at 2 weeks) and PDX2 (mean = 12.1 ng/mL at 3 weeks vs. 0 ng/mL at 2 weeks) cell lines, which presented an increase in VN secretion to media after three weeks of culture, unrelated to greater cell growth (Figure 1C). SK-N-BE(2) and SH-SY5Y cell lines showed a larger secretion of VN per million cells when cultured in HGs without added VN (mean = 16.3 ng/mL and 12.1 ng/mL, respectively) compared to HGs with added VN (mean = 13.5 ng/mL and 7.8 ng/mL, respectively) at both two and three weeks of culture, despite the increasing trend in cell growth observed in the HGs with added VN, which was more notable in the HGs with three weeks of culture (Figure 1C).

### 2.3. Clinical and Genetic Characteristics of NB Patients Included in This Study

Having detected VN in the culture media of the studied aggressive NB cell lines, we next sought to analyze whether the same would occur in the plasma of NB patients. A total of 114 NB patients aged between 0 and 296 months at time of diagnosis (mean = 27.2 ± 35.2) were included in this study, of whom 52 were older than 18 months and 54 showed metastasis at diagnosis. Homogeneous *MYCN* amplification was detected in twenty and heterogeneous amplification in four NB patients. Typical SCAs (typSCAs) of NB patients were detected in 60 cases, two tumors presented atypical SCAs (atypSCAs) and 33 NB patients showed numeric chromosomal aberrations (NCAs). After 5–204 months of follow-up (mean = 65.1 ± 37.8), 81 patients remained alive (Table 1, Figure S1).

### 2.4. Discriminatory Power of Plasma VN Levels for NB Patient Outcomes

**Figure 2:**
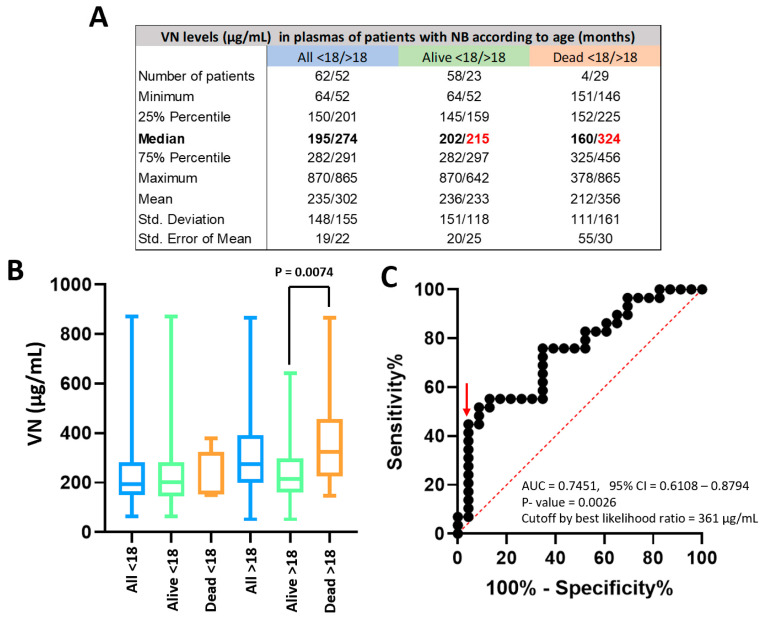
VN levels in plasma of NB patients. (**A**) Descriptive statistics of patient numbers and VN levels detected by ELISA in the plasma of all patients younger and older than 18 months of the patients who remained alive and of those who died. Median VN values are highlighted in bold format and the red font points out the lower levels of VN in alive than in dead patients older than 18 months. (**B**) Graphic representation of VN levels in the mentioned groups. VN concentration was significantly different between alive and dead patients older than 18 months (*p*-value = 0.0074). (**C**) The ROC curve obtained for patients older than 18 months was significant (*p*-value = 0.0026) and allowed us to establish a cutoff of 361 µL/mL (red arrow), over which patients showed a poor prognosis.

VN levels in the plasma of the 33 NB patients who died were significantly higher than those obtained in the 81 patients who survived (median = 308 µg/mL vs. 205 µg/mL, respectively, *p* = 0.0009, Figure S2). In line with the prognostic power of this factor in NB patients, however, age showed a pronounced association with death and is the confounder; only four of the 62 patients younger than 18 months died after the abovementioned follow-up, without significant differences in their plasma VN concentration (Figure 2A,B). Plasma VN levels in NB patients older than 18 months who died (N = 29) were significantly higher than in the ones that survived [N = 23] (median = 324 µg/mL vs. 215 µg/mL, respectively, *p* = 0.0074, Figure 2A,B). As plasma VN levels had outcome discriminatory power in those patients, a ROC curve was represented to identify the optimal cutoff according to survival (Figure 2C). The ROC curve was significant (*p* = 0.0026) with AUC = 0.75 (95% CI = 0.61–0.88), and a cutoff of 361 µg/mL showed the best likelihood ratio (10.31). The mean VN concentration previously described in the plasma of healthy controls with similar ages was 367.7 µL/mL [37], so the cutoff is also almost over this value (Figure S3).

**Figure 3:**
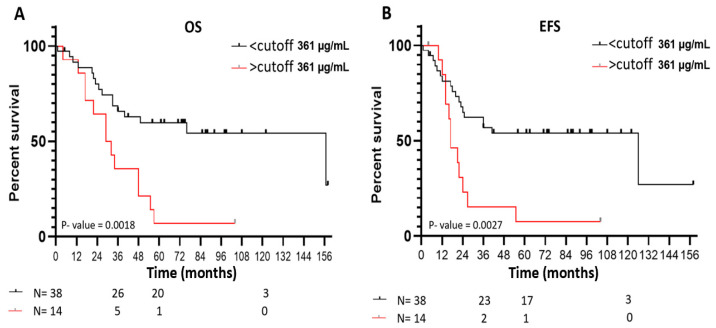
Survival probabilities of NB patients older than 18 months according to VN levels. Kaplan–Meier curves for (**A**) overall survival (OS) and (**B**) event-free survival (EFS) reflected the significantly lower survival of NB patients with VN plasma levels over the cutoff (*p* = 0.0018 and *p* = 0.0027, respectively). Five-year OS for patients with VN levels under and over the cutoff was 53% and 7%, respectively, and EFS was 45% and 7%, respectively.

Kaplan–Meier curves for overall survival (OS) and event-free survival (EFS) reflected the significantly worse outcome of NB patients older than 18 months with VN plasma levels over the cutoff compared to the patients with VN levels under these values (*p* = 0.0018 and *p* = 0.0027, respectively) (Figure 3A,B). Five-year OS was 7% for patients with VN levels over the cutoff and 53% for those under, and five-year EFS were 7% and 45%, respectively.

### 2.5. Plasma VN Level Association with Stage and Genetic Features of NB

Besides the association of plasma VN levels with outcomes in NB patients older than 18 months, we also studied the potential link of VN concentrations with certain clinical and genetic factors (Table 2). We detected an increase in the amount of patients with metastatic disease at diagnosis and VN levels over the cutoff (*p* = 0.021), and of NB with SCAs (*p* = 0.014), specifically with the typical loss of 11q (−11q, *p* = 0.006).

Cox regression analysis using stepwise forward and backward Wald methods proved that the cutoff VN level of 361 µg/mL had a significant outcome predictive value, independent of other clinical and genetic factors (Table 3).

## 3. Discussion

The search for new biomarkers that improve and speed up patient diagnosis, better classify them into risk groups, and define new therapeutic strategies is one of the greatest challenges of oncological research in recent years. Circulating biomarkers have the added advantage of being non-invasive compared to biomarkers analyzed on tumor biopsies. Circulating biomarkers currently used in NB diagnosis and prognosis include urine catecholamines, serum lactate dehydrogenase, ferritin, and neuron-specific enolase [38]. Circulating tumor cells (e.g., cell markers such as GD2), specific genetic alterations in cell-free DNA (e.g., *MYCN, ALK*, C-Circles), mRNAs (e.g., *TH, PHOX2B* and *DCX*), and more recently, hypermethylation (e.g., *RASSF1A*) and extracellular vesicles, also seem of interest for defining prognosis and detecting early relapses and minimal residual disease [38,39,40,41]. These biomarkers are related to the underlying mechanisms influencing NB progression, as an impairment of cell cycle regulation mediated by *MYCN* and *ALK* targets [42,43]; telomere maintenance mechanisms, especially alternative telomere elongation, which maintains undefined cell survival and division [41,44]; and neuroblast plasticity cause a partial transition from noradrenergic to migratory and chemoresistant mesenchyme cells [45,46]. Interestingly, these biomarkers are currently sought not only at the level of malignant cells but also in the tumor microenvironment, which along with its important role in tumor progression and metastasis also represents a whole universe of therapeutic targets [40,47,48]. The peculiarities of the tumor microenvironment and especially of the ECM play a key role in clonal selection [23,46,48] and are partly responsible for the different mutation landscape crucial in tumor evolution and resistance to therapies [49]. ECM features, such as a high presence of cross-linked reticuline fibers, low amount of glycosaminoglycans, high quantity of sinusoidal blood and lymphatic vessels and a high expression of territorial VN, have been associated with tumor aggressiveness and poor prognosis [5,50].

In previous studies, we proposed VN as a tissue biomarker based on its high expression by IHC in NB biopsies and several experimental models [5,22,23,24,25,26]. In this work, we went one step further to study whether the NB cells grown in our new 3D models secrete VN to culture media and whether secretion can be detected in the plasma of NB patients.

VN release from NB cells to culture media was higher in 3D models than in monolayer cultures. This reflects the importance of using biomimetic models which reflect not only cell–cell interactions but also cell–scaffold communication. In this sense, although we linked high concentrations of VN to HGs, cells continued to secrete their own VN at considerable levels (although somewhat lower than those of HGs without added VN). A previous study with glioma cells showed an increase in cell migration when cells were cultured with complete human serum, as well as a reduction of motility when the VN of those serums was depleted [32]. Similar effects were described in other publications with several cancer cells, and the authors postulated that VN present in plasma is a key migration-inducing factor and more potent chemoattractant than already known pro-metastatic chemokines or growth factors [33,51]. Moreover, VN glycosylation patterns can impact its ability to interact with ligands and induce advantageous conformational changes, as has been shown in human hepatocellular carcinoma-derived plasmas in comparison to healthy controls, as well as in ovarian cancer ascites [52,53]. Interestingly, no relationship was found between the number of cells and amount of VN secretion in our NB models. Hao et al. described high VN levels in the serum of breast cancer patients compared with healthy controls, and they also pointed to a negative correlation between tumor size and serum VN levels. Their hypothesis was that this could be due to a greater secretion of proteases (such as MMP2) which degrade VN upon tumor progression [54]. Whether this could apply in our 3D models (and to what extent) or whether this reduction in VN secretion could be due to cellular activity focused on other cellular functions, to differences in synthetized VN retention between cells or in scaffolds, or to reduced viability caused by cell overgrowth are also interesting questions for future studies.

High VN levels in plasma have been previously described in glioma, melanoma, hepatic, ovary, endometrium and breast cancer patients associated with poor prognosis [12,14,15,16,29,30,31,32,33]. Children and adolescents with Hodgkin lymphoma who relapsed and pediatric patients with acute lymphoblastic leukemia also showed increased levels in plasma of this glycoprotein [27,28]. Nonetheless, to our best knowledge, only one publication has studied VN plasma levels in pediatric patients with solid tumors, without obtaining significant differences with healthy controls. Only eight plasmas from eight children with NB were included in that publication: one from an initial stage patient and seven from children in advanced stages of illness [34]. The small number of plasmas precluded studying the association of VN levels with tumor aggressiveness and prognosis. Our study includes 114 plasmas from NB patients, a not inconsiderable figure taking into account the small number of annual cases of this rare cancer type. We observed that high plasma VN levels had a prognostic value, independently from clinical and genetic factors of NB applying Cox regression. These findings substantialize plasma VN as a potential circulating prognostic biomarker for NB in patients aged over 18 months. However, mean plasma VN values obtained in NB patients were lower than those reported in healthy controls [37] of the same age (269.5 µg/mL vs. 367.6 µg/mL, respectively; Supplementary Figure S3). As the ELISA kits used for VN detection were different, there may be discrepancies between the values obtained, as has been observed in studies comparing healthy control levels [29,37,55].

High VN expression was observed by our group in ECM of HR-NB biopsies [5] with significant branching in VN patterns, which seemed to form tracks enabling malignant cells to reach blood vessels [22]. VN could intervene in cell adhesion, proteinase secretion and tissue remodeling to achieve a more favorable ECM, leading to tumor progression, migration and survival. In this context, VN has been shown to activate phosphorylation of p42/44 MAPK and AKT in cell lines, and VN serum has been correlated with PI3K and AKT levels [15,33]. Moreover, VN is considered to play an important role in the regulation of endothelial permeability and VEGF signaling, promoting angiogenesis and vascular permeability [9]. Once in the bloodstream and in other fluids, such as lymph, ascites and cerebrospinal fluid, VN could promote circulating tumor cells to disseminate and adhere to distal organs [54,56]. As VN can be produced in the peritoneal cavity, liver, and bones [51,57,58], these could represent suitable premetastatic niches for forming new NB metastatic tumors.

## 4. Materials and Methods

### 4.1. Two-Dimensional Cell Cultures

SK-N-BE(2) and SH-SY5Y NB cell lines were acquired from American Type Culture Collection (ATCC, Masassas, VA, USA). Both cell lines were expanded in IMDM medium (Gibco, Life Technologies, Waltham, MA, USA) supplemented with 10% FBS, 1% insulin/transferrin and 1% Penicillin/streptomycin at 37 °C in 5% CO_2_ atmosphere. Patient-derived xenograft 1 (PDX1) and PDX2 cells were previously established as described in [36]. Both cell lines were expanded in DMEM-high glucose GlutaMAX™ medium, supplemented with 1% B-27™ without Vitamin A (Gibco, Life Technologies, Waltham, MA, USA), 20 ng/mL of EGF and FGF, and 1% Penicillin/streptomycin. Culture medium was replaced every 2–3 days.

### 4.2. Three-Dimensional Hydrogel Construction

Three-dimensional HGs were based on previous work [59]. Briefly, sf (Sigma Aldrich, Merck, USA) was mixed with previously synthetized and lyophilized GTA (Sigma Aldrich, Merck, St. Louis, MO, USA) in unsupplemented IMDM cell culture medium to obtain 4% *w*/*v* solutions. The ratio of sf/GTA was 75:25. VN (PrepoTech, Rocky Hill, NJ, USA) resuspended in dPBS was added to half of the solution, with a final concentration of 0.4 mg/mL. dPBS was added in the remaining solution to construct HGs without VN. Horseradish peroxidase (20 U/mL) was also added to the mix. Prior to gelification, 1.25 × 10^5^ of commercial NB cells, or 2.5 × 10^5^ of PDX cells, previously cultured in 2D, were resuspended in the solution per each HG. To start the polymerization process, 2 µL of hydrogen peroxide (0.01%) was placed in the center of each well in a 24-well plate and mixed by fast and smooth pipetting with aliquots of 60 μL of the mix solution. HGs were incubated at 37 °C for 1 h to complete gelification. Subsequently, 2 mL of growth culture medium was added. Culture media were replaced and collected for ELISA every three days. We kept the 3D models for two and three weeks.

### 4.3. Patients and Samples

Blood plasmas from 114 NB patients diagnosed between 2003 and 2023 were included in this study. Peripheral blood samples were collected at the time of primary tumor biopsies. All samples were sent to the Spanish Reference Centre for NB Molecular and Pathological studies (Department of Pathology, University of Valencia-INCLIVA) and stored in our biobank (reference B.0000339 29/01/2015). Histopathological data of the NBs were provided by the reference group pathologist. Clinical data were provided by the attending pediatric oncologist when possible, or by the Reference Centre for NB Clinical Studies. These included outcome data of EFS (defined as length of time from diagnosis to any progression, death or to the date of last contact) and OS (defined as length of time from diagnosis until death or last medical check-up in surviving patients). Genetic data were compiled from our internal database NeuPAT [60]. All clinical-biological data are shown in Table 1. This study was approved by the Research Ethics Committee of the Clinic Hospital of Valencia (No. 2020/025, Act: 372, 30 September 2021). Participants or their family members/legal guardians provided written informed consent for the research studies performed in our laboratory.

### 4.4. Plasma VN Determination

Peripheral blood samples of NB patients were collected in sterile EDTA tubes. Samples were centrifuged at 2000× *g* for 10 min to separate plasma. Plasma samples were stored at −80 °C until assayed. VN levels were measured using a sandwich colorimetric enzyme-linked immunosorbent assay kit (Human Vitronectin ELISA Kit, Novus Biologicals, Centennial, CO, USA) technique according to the manufacturer’s instructions. Briefly, the human VN ELISA kit (Novus Biologicals, LLC 10771 E Easter Ave Centennial, CO 80112, USA) recognizes both recombinant and natural human VN with an assay sensitivity of 15.19 pg/mL. Samples were loaded and incubated with biotinylated antibody, and HRP reaction, absorbance reading and results calculation steps were performed using a Triturus automated analyzer (Grífols, Barcelona, Spain). To bring VN levels within the detection ranges of the kit, plasma samples of patients were diluted between 1:1000 and 1:5000 before assay. For VN level determination in media of 2D cell cultures, 8 mL of medium from confluent T75 cell culture flasks were centrifugated at 1200 rpm for 5 min to remove cellular debris. Next, media were concentrated 16 times with SpeedVac™ (Thermo Fisher Scientific, Newington, NH, USA). The number of cells of 2D cultures were determined by a TC20 Automated Cell Counter (Bio Rad, Hercules, CA, USA) after mixing with trypan blue for comparison with the VN level secreted to culture media. For 3D cell cultures, media were collected from two HG replicates (4 mL) together, for each composition and culture time. Next, they were centrifugated as before and concentrated eight times with SpeedVac™. With this step, we reduced the variability intrinsic to HG replicates and achieved the minimum VN level for ELISA detection. As culture media was collected with each change (every 3 days), we analyzed VN levels with ELISA at several time points. However, in this study, VN concentration was obtained from culture media of the two HGs together, relative to the total cell count from the same two HGs at the 2-week and 3-week time points (Figure 1C, no error bars applicable). The HGs were formalin-fixed and paraffine-embedded at 2 and 3 weeks, and a 3 µm section was stained with hematoxylin and eosin. Slides were scanned with Ventana iScan (Roche, Basel, Switzerland). The number of cells per section was determined with StarDist extension for QuPath and scaled to the total volume of HGs (28 mm^3^). The results were multiplied by the dilution or concentration factor, and those from culture media were also scaled to the number of cells (per millions) in the cultures.

### 4.5. Statistical Analysis

Statistical analysis was carried out using GraphPad Prism 8 (Graphpad Software, Boston, MA, USA) and SPSS 28.0 (SPSS Inc., Chicago, IL, USA). The Kolmogorov–Smirnov test was carried out to investigate data distribution. Non-paired T-tests were applied to compare VN release in 2D and 3D cultures, and Pearson’s r correlation was used to compare VN secretion to culture media and HGs cell count. For patient cohort analysis, Mann–Whitney and Kruskal–Wallis tests were applied to compare survival and plasma VN levels between the age groups, while Spearman’s r correlation tests were performed to study associations between plasma VN levels and patients’ clinical/molecular features. The receiver operating characteristic (ROC) curve was performed to assess discriminatory ability with an estimated area under curve (AUC). A suitable cutoff value, based on the best likelihood ratio, was selected from ROC curves to obtain the optimal sensitivity and specificity. Survival curves were made using the Kaplan–Meier method, and Cox regression analysis was employed to assess OS- and EFS-related predictors (Wald forward and backward stepwise methods). Patient age at diagnosis was the confounding variant (age > 18 months old at diagnosis is associated with poor prognosis [19], and healthy controls of more than 12 months had more VN in plasma than younger controls [37]); therefore, only patients > 18 months old were included in ROC, Kaplan–Meier and Cox analysis.

## 5. Conclusions

This study and our previous research with patient biopsies and other experimental models all suggest that VN (expressed by tumor cells and detected in plasma) could be closely linked to tumor aggressiveness and poor prognosis in NB patients, appearing to play a role in different steps of cancer progression. Herein, we provide a proof of principle of VN secretion by malignant neuroblasts as a novel predictive circulating biomarker. Further work is needed to refine the estimation of the reference VN. Collaborative studies including more plasma samples from patients and healthy controls must be conducted for VN levels to be considered alongside classical prognosis factors in therapeutic decision making, which would represent a step forward in precision medicine. In combination with classic therapies, drugs such as Cilengitide [61,62,63,64] (which binds to integrins, acting as a competitive inhibitor of their binding to VN and is currently being tested by our group in 3D models) and fibrinogen analogs (which seem to quench VN chemoattraction [33,51]) could bring a significant improvement regarding the survival of patients with NB and other cancers.

## Figures and Tables

**Table 1:** Patient cohort characteristics and tumor features, grouped by relationship with VN plasma level.

		Total Cohort			VN > 361 µg/mL			VN < 361 µg/mL		
		Mean ± SD	N	%	Mean ± SD	N	%	Mean ± SD	N	%
Total			114	100%		24	21%		90	79%
**OS**	Dead	65.1 ± 37.8	33	29%	53.4 ± 31.0	14	58%	68.3 ± 38.9	19	21%
	Alive		81	71%		10	42%		71	79%
**EFS**	Event	52.7 ± 39.7	46	40%	46.0 ± 53.4	14	58%	54.5 ± 40.6	32	36%
	Non-event		68	60%		10	42%		58	64%
**Age**	>18 months	27.2 ± 35.2	52	46%	39.4 ± 57.7	14	27%	23.9 ± 25.2	38	73%
	≤18 months		62	54%		10	16%		52	84%
**Stage**	Stage 4		54	47%		15	63%		39	43%
	Non-stage 4		58	51%		9	38%		51	57%
	Unknown		2	2%		2	8%		0	0%
***MYCN*** **Status**	Amplified		20	18%		6	25%		14	16%
	Het-amplified		4	4%		0	0%		4	4%
	Non-amplified		90	79%		18	75%		72	80%
**Chromosomic profile**	Typical SCA		60	53%		17	71%		43	48%
	Atypical SCA		2	2%		1	4%		1	1%
	NCA		33	29%		3	13%		30	33%
	Unknown		19	17%		3	13%		16	18%
**−1p**	Present		29	25%		8	33%		21	23%
	Absent		66	58%		13	54%		53	59%
	Unknown		19	17%		3	13%		16	18%
**+1q**	Present		9	8%		3	13%		6	7%
	Absent		86	75%		18	75%		68	76%
	Unknown		19	17%		3	13%		16	18%
**+2p**	Present		23	20%		7	29%		16	18%
	Absent		72	63%		14	58%		58	64%
	Unknown		19	17%		3	13%		16	18%
**−3p**	Present		13	11%		5	21%		8	9%
	Absent		82	72%		16	67%		66	73%
	Unknown		19	17%		3	13%		16	18%
**−4p**	Present		10	9%		2	8%		8	9%
	Absent		85	75%		19	79%		66	73%
	Unknown		19	17%		3	13%		16	18%
**−11q**	Present		29	25%		12	50%		17	19%
	Absent		66	58%		9	38%		57	63%
	Unknown		19	17%		3	13%		16	18%
**−17q**	Present		49	43%		14	58%		35	39%
	Absent		46	40%		7	29%		39	43%
	Unknown		19	17%		3	13%		16	18%

OS, overall survival; EFS, event-free survival; Het-amplified, heterogeneously amplified; SCA, segmental chromosomal alterations; NCA, numerical chromosomal alteration. OS, EFS, and age means and standard deviations (SDs) are expressed in months.

**Table 2:** VN level by cutoff is associated with death, stage, genomic profile, and 11q loss in NB patients older than 18 months.

	Correlation of Clinic-Molecular Features of NB Patients Older than 18 Months and VN Levels			
	Plasma VN Levels		Spearman r (95% CI)	*p*-Value (Two-Tailed)
	>361 µg/mL	<361 µg/mL		
**Death (yes/no)**	13/1	16/22	0.363 (0.186–0.517)	0.000 *
**Relapse (yes/no)**	13/1	18/20	0.179 (−0.010–0.356)	0.1572
**Unknown/metastatic/localized**	1/10/3	23/15	0.217 (0.003–0.392)	0.021 *
**MYCN status (MNA/MNNA)**	4/10	11/27	0.115 (−0.077–0.298)	0.226
**Genomic profile (unknow/SCAs/NCAs)**	2/12/0	6/29/3	0.248 (0.046–0.430)	0.014 *
**−1p (unknown/present/absent)**	2/6/6	7/17/14	0.157 (−0.050–0.351)	0.125
**+1q (unknown/present/absent)**	2/2/10	8/6/24	−0,005 (−0.211–0.201)	0.959
**+2p (unknown/present/absent)**	2/5/7	7/11/20	0.138 (−0.073–0.330)	0.192
**−3p (unknown/present/absent)**	2/4/8	8/7/23	0.126 (−0.083–0.324)	0.222
**−4p (unknown/present/absent)**	2/2/10	8/7/23	−0.024 (−0.229 to 0.183)	0.819
**−11q (unknown/present/absent)**	2/9/3	7/16/15	0.277 (0.076–0.456)	0.006 *
**+17q (unknown/present/absent)**	2/9/3	8/22/8	0.176 (−0.032–0.369)	0.087

*MYCN* status: MNNA, *MYCN* non-amplified; *MYCN* heterogeneously amplified; MNA, *MYCN* amplified; NCA, numerical chromosomal alteration; SCA, segmental chromosomal alterations. Significant *p*-values (<0.05) are highlighted in bold format and with asterisks. The *p*-value that indicates the significant association of VN level and death is highlighted in red.

**Table 3:** Cox regression tests results after applying stepwise forward and backward Wald methods: last step.

Variable	B	SE	Wald	Exp (B)	*p*-Value
**OS Wald (forward stepwise) method**					
VN cutoff	1.329	0.447	8.844	3.778	0.003 *
**OS Wald (backward stepwise) method**					
VN cutoff	1.263	0.449	7.922	3.536	0.005 *
*MYCN* status	0.840	0.460	3.335	2.316	0.068
**EFS Wald (forward stepwise) method**					
−4p	−1.471	0.752	3.825	0.230	0.050
VN cutoff	1.460	0.472	9.575	4.307	0.002 *
**EFS Wald (backward stepwise) method**					
−4p	−1.471	0.752	3.825	0.230	0.050
VN cutoff	1.460	0.472	9.575	4.307	0.002 *

VN levels dichotomized by cutoff in NB patients older than 18 months, *MYCN* status (*MYCN* status: MNNA, *MYCN* non-amplified; hetMNA, *MYCN* heterogeneously amplified; MNA, *MYCN* amplified) and loss of 4p chromosomic region were the variables maintained in the last step of the Cox regression tests according to EFS and OS; however, only VN levels showed significant *p*-values. Other variables without significant results included in the Cox regression analysis were age, stage (localized/metastatic), genomic profile (NCAs, chromosomal numeric aberrations/SCAs, chromosomal segmental aberrations) and typical SCAs (−1p, +1q, +2p, 3p−, −4p, −11q, +17q). B: beta coefficient; SE: standard error. Coefficients Exp (B)  >  1 indicate that high values of this parameter increase its probability of being an independent poor prognostic factor. *: Significant *p*-values (< 0.05).

## Data Availability

The datasets used and analyzed in the current study are available from the corresponding author upon reasonable request.

## Supplementary Materials

The following supporting information can be downloaded at: https://www.mdpi.com/article/10.3390/ijms25168733/s1.
