# Supplementary material for: Vitronectin Levels in the Plasma of Neuroblastoma Patients and Culture Media of 3D Models: A Prognostic Circulating Biomarker?
Source: Int J Mol Sci. 2024 Aug 10;25(16):8733. doi: 10.3390/ijms25168733 (PMC11354570; doi:10.3390/ijms25168733)
Supplement: Supplementary file 1 [file ijms-25-08733-s001.zip › ijms-3122857-supplementary.pdf]

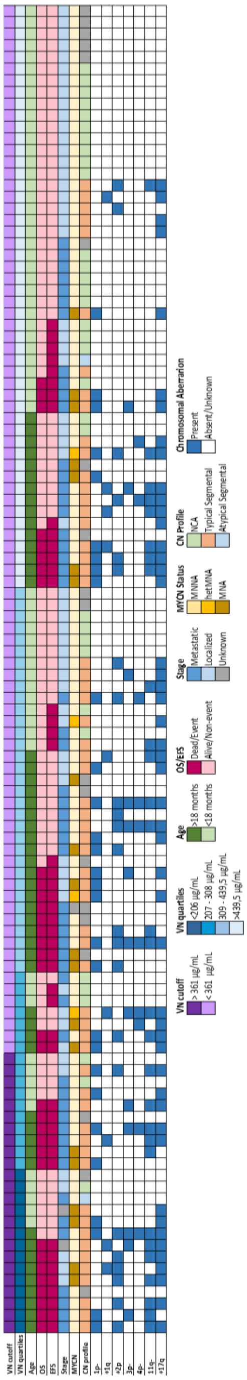

Figure S1. Plasma VN level of NB cohort and clinic-molecular landscape overview. The top panels show VN levels in plasma stratified by the cutoff of 361 μg/mL in patients older than 18 months, and by quartiles across all dead patients. The central panels show clinical characteristics including age, overall survival (OS), event free survival (EFS) and stage. MYCN status, genomic profile and typSCAs are shown in the bottom panels. See figure legend for color codes. MNNA, MYCN non-amplified; hetMNA, MYCN heterogeneously amplified; MNA, MYCN amplified; NCA, numerical chromosomal alteration; TypSCA, typical segmental chromosomal alterations; AtypSCA, atypical segmental chromosomal alterations.

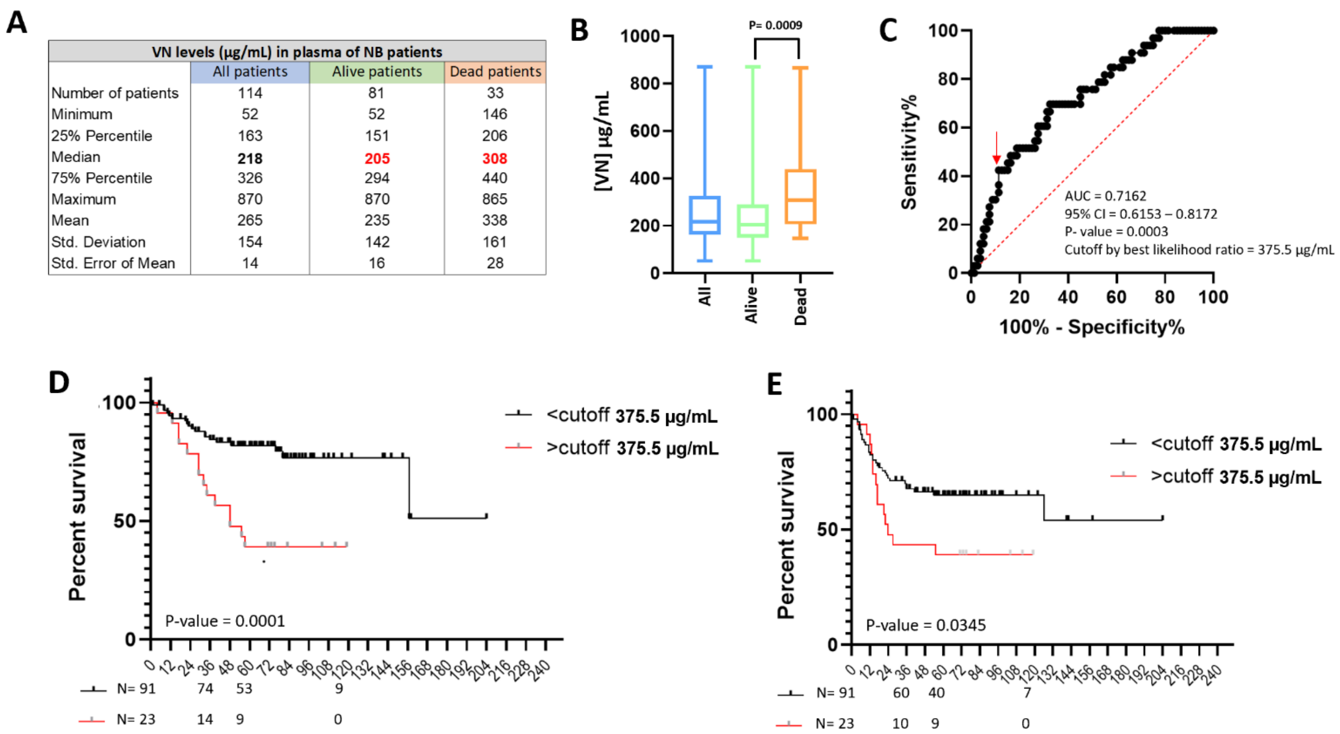

VN levels (µg/mL) in plasma of NB patients	All patients	Alive patients	Dead patients
Number of patients	114	81	33
Minimum	52	52	146
25% Percentile	163	151	206
Median	**218**	**205**	**308**
75% Percentile	326	294	440
Maximum	870	870	865
Mean	265	235	338
Std. Deviation	154	142	161
Std. Error of Mean	14	16	28

Figure S2. VN levels in plasma of all NB patients. A. Descriptive statistics of number of patients and VN levels detected by ELISA in the plasma of all NB patients, patients who remained alive and those who died. B. Graphic representation of VN levels in the abovementioned groups. VN concentration was significantly different between alive and dead patients (p-value = 0.0009). C. The ROC curve obtained for all NB patients was significant (p = 0.0003), allowing us to establish a cutoff of 375.5 µL/mL (red arrow) over which patients showed a poor prognosis. However, only four patients younger than 18 months died and VN levels were not significant for these patients (figure 3A), so we decided to focus on NB patients older than 18 months and to use the cutoff obtained for those plasmas (Figure 3C). Kaplan-Meier curves for D. OS and E. EFS probabilities reflected the significantly low survival of NB patients with VN plasma levels over the cutoff (p = 0.0001 and p = 0.0345, respectively). Five-years OS probability for patients with VN levels under and over the cutoff was 58% and 39% respectively, and for EFS was 44% and 39%, respectively.

A

VN levels (µg/mL) in plasmas of patients with NB according to age range (months)			
	All <12/13-60/>60	Alive <12/13-60/>60	Dead <12/13-60/>60
Number of patients	48/6/10	44/34/2	4/22/2
Minimum	73/52/200	73/52/200	151/146/240
25% Percentile	151/167/244	148/149/200	152/208/245
**Median**	**195/230/299**	**202/211/249**	**160/316/378**
75% Percentile	286/337/442	286/292/297	325/453/524
Maximum	870/865/644	870/642/297	378/865/644
Mean	242/152/351	245/220/249	212/346/388
Std. Deviation	156/152/141	160/121/69	111/165/154
Std. Error of Mean	22/20/45	24/21/49	55/35/58
**VN levels (µg/mL) in plasmas of healthy controls according to age range (months)***			
Minimum	185/202/227		
Mean	293/367/334		
Maximum	420/651/562		

B

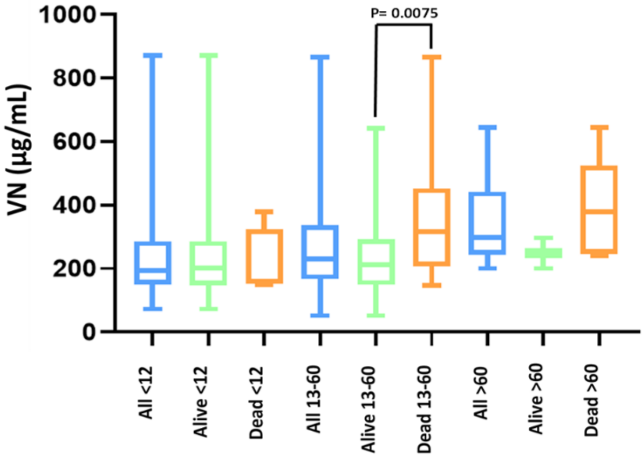

Figure S3. VN levels in plasma of NB patients stratified by age. A. Descriptive statistics of number of patients and VN levels detected by ELISA in plasma of all patients younger than 12 months, between 13 and 60 months and older than 60 months, of the patients who remained alive and of those who died with the same age ranges. *Comparation with VN levels described in plasma of healthy children with the same age ranges[37]. B. Graphic representation of VN levels in the mentioned groups. VN concentration between alive and dead patients with 13-60 months old was significant (p-value = 0.0075).
